# Spatial Distribution of Macrophage and Lymphocyte Subtypes within Tumor Microenvironment to Predict Recurrence of Non-Muscle-Invasive Papillary Urothelial Carcinoma after BCG Immunotherapy

**DOI:** 10.3390/ijms25094776

**Published:** 2024-04-27

**Authors:** Julius Drachneris, Mindaugas Morkunas, Mantas Fabijonavicius, Albertas Cekauskas, Feliksas Jankevicius, Arvydas Laurinavicius

**Affiliations:** 1 Department of Pathology and Forensic Medicine, Institute of Biomedical Sciences, Faculty of Medicine, Vilnius University, 03101 Vilnius, Lithuania; julius.drachneris@vpc.lt; 2 National Center of Pathology, Affiliate of Vilnius University Hospital Santaros Klinikos, 08406 Vilnius, Lithuania; 3 Clinic of Gastroenterology, Nephrourology and Surgery, Institute of Clinical Medicine, Faculty of Medicine, Vilnius University, 08406 Vilnius, Lithuania; 4 Center of Urology, Vilnius University Hospital Santaros Klinikos, 08406 Vilnius, Lithuania

**Keywords:** tumor microenvironment, digital image analysis, tumor-associated macrophages, tumor-infiltrating lymphocytes, non-muscle-invasive bladder cancer

## Abstract

Non-muscle-invasive papillary urothelial carcinoma (NMIPUC) of the urinary bladder is the most common type of bladder cancer. Intravesical Bacille Calmette–Guerin (BCG) immunotherapy is applied in patients with a high risk of recurrence and progression of NMIPUC to muscle-invasive disease. However, the tumor relapses in about 30% of patients despite the treatment, raising the need for better risk stratification. We explored the potential of spatial distributions of immune cell subtypes (CD20, CD11c, CD163, ICOS, and CD8) within the tumor microenvironment to predict NMIPUC recurrence following BCG immunotherapy. Based on analyses of digital whole-slide images, we assessed the densities of the immune cells in the epithelial–stromal interface zone compartments and their distribution, represented by an epithelial–stromal interface density ratio (IDR). While the densities of any cell type did not predict recurrence, a higher IDR of CD11c (HR: 0.0012, *p*-value = 0.0002), CD8 (HR: 0.0379, *p*-value = 0.005), and ICOS (HR: 0.0768, *p*-value = 0.0388) was associated with longer recurrence-free survival (RFS) based on the univariate Cox regression. The history of positive repeated TUR (re-TUR) (HR: 4.93, *p*-value = 0.0001) and T1 tumor stage (HR: 2.04, *p*-value = 0.0159) were associated with shorter RFS, while G3 tumor grade according to the 1973 WHO classification showed borderline significance (HR: 1.83, *p*-value = 0.0522). In a multivariate analysis, the two models with a concordance index exceeding 0.7 included the CD11c IDR in combination with either a history of positive re-TUR or tumor stage. We conclude that the CD11c IDR is the most informative predictor of NMIPUC recurrence after BCG immunotherapy. Our findings highlight the importance of assessment of the spatial distribution of immune cells in the tumor microenvironment.

## 1. Introduction

Bladder cancer (BC) is the 12th most prevalent cancer worldwide [1]. While total cystectomy is the treatment of choice in muscle-invasive BC (MIBC), non-muscle-invasive bladder cancer (NMIBC) is usually treated with transurethral resection (TUR) [2]. For patients with intermediate and high-risk tumors, adjuvant intravesical Bacille Calmette–Guérin (BCG) immunotherapy is administered to reduce the risk of relapse [2]. However, despite treatment, over 30% of patients still experience a relapse, and a significant proportion of them experience tumor progression to muscle-invasive disease [3]. Therefore, an early shift to other, more aggressive treatment strategies in selected patients with refractory disease may improve patient outcomes [4].

The tumor microenvironment plays an important role in the tumor response to immunotherapy. In particular, the course of NMIBC in the context of BCG immunotherapy can potentially be predicted by relevant biomarker modeling [5]. Several constituents of the immune tumor microenvironment have been reported as being prognostic in NMIBC: tumor-associated macrophages (TAMs) [6,7,8,9,10,11,12,13], tumor-infiltrating lymphocytes (TILs), NK cells [7], dendritic cells [11,14], and eosinophils [15,16].

Hanada et al. first explored the importance of TAMs and microvasculature density in BC and found the higher density of TAM to be associated with muscle-invasive disease, vascular invasion, higher metastasis rate, and poor survival [6]. Further studies reported higher densities of TAMs to be associated with stromal invasion [7], failure of BCG immunotherapy [8], a higher rate of recurrence [8,9,13], and progression to MIBC [10,11]. Interestingly, Ajili et al. found the higher ratio of CD68+ macrophage densities within the epithelial versus stromal compartments to be associated with better recurrence-free survival (RFS) [9]. Subsequent studies revealed the importance of macrophage polarization toward M1 macrophages (pro-inflammatory) and M2 macrophages (anti-inflammatory): higher M1-like macrophage densities were associated with better RFS [12], while high M2-like macrophage densities were associated with worse RFS [8,12].

TILs and their distribution patterns within the tumor microenvironment have been extensively studied as important predictors of patient outcomes in various tumors, along with efforts to standardize their assessment [17,18,19]. In NMIBC studies focusing on TIL subclasses, Th2 cell density was predictive of response to BCG immunotherapy [15,16], while a high density of regulatory T cells was associated with shorter RFS [13]. CD8+ TILs were associated with tumor stage [20] and were co-localized with PD-L1-expressing cells [21]; however, their density in the tissue of NMIBC did not correlate with survival probability [8,21,22]. Bieri et al. [23] presented a modified immunoscore obtained by image analysis of tissue microarray images of CD3, CD8, and CD45RO which were associated with longer progression-free survival in the high-risk patient group. We have recently reported [24] that a high ratio between CD8+ cell densities in the intraepithelial and stromal compartments was an independent predictor of longer RFS in the cohort of NMIPUC patients treated with BCG immunotherapy, thus supporting the importance of the spatial distribution of CD8+ lymphocytes in the host response to antitumor immunity. The prognostic significance of inducible co-stimulator (ICOS)-positive lymphocytes in the context of immunotherapy has been demonstrated in several tumor types [25], including BC [26]. However, this biomarker has not been investigated in NMIBC patients treated with intravesical BCG so far.

Tertiary lymphoid structures (TLSs) are observed in various tumors and, as a constituent of the antitumor milieu, are prognostic of patient outcomes [27]. Studies investigating TLSs in BC have found a more common incidence of TLSs (75%) in MIBC in comparison to NMIBC (25%) [28] and different constitutions of TLSs in patients not responding to checkpoint inhibitor therapy [29]. Data on the significance of TLSs in the context of BCG immunotherapy are lacking.

Non-muscle-invasive papillary urothelial carcinoma (NMIPUC) is the most common type of NMIBC, with a specific histologic architecture defined by the formation of papillary structures. We have narrowed our patient selection to NMIPUC cases to assess spatial distribution in the context of this specific tumor histologic architecture and to obtain a more homogenous patient cohort. In this retrospective study, we explore the prognostic value of CD20, CD11c, CD163, ICOS, and CD8-positive cells and their spatial distributions in the NMIPUC microenvironment of 155 patients treated with BCG immunotherapy.

## 2. Results

### 2.1. Exploring the Interface Zone Width Settings to Optimize Predictive Indicators

**Figure 1:**
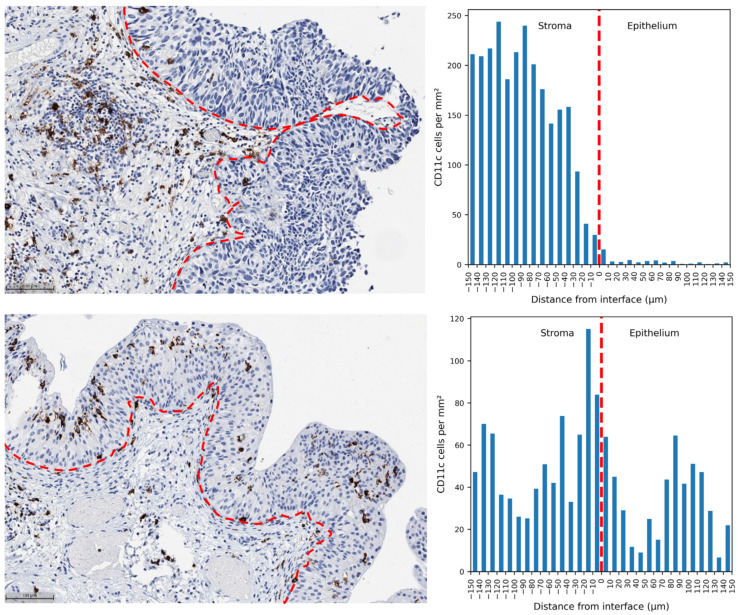
CD11c-positive cell infiltration density profiles in the NMIPUC of two selected patients to illustrate different infiltration patterns. Upper panel, patient A with predominant infiltration in the tumor stroma (IDR = 0.074): a representative IHC image area (**left**) and a bar plot (**right**) representing the CD11c cell density distribution across the interface. Lower panel, patient B with relatively higher CD11c cell density in the epithelium (IDR = 0.424). The red dotted line represents the epithelial–stromal interface. A 100 µm measure is added for the reference. Negative distances in the plot represent the stromal aspect of the interface zone. For CD163, CD20, ICOS, and CD8 IHC images in patients A and B, see Supplementary Figures S1 and S2.

Immunohistochemical (IHC) assessment of tumor tissue by using digital image analysis revealed variable patterns of the immune cell distributions at the epithelial/stromal interface. They ranged from cases with predominant infiltration in the stroma represented by a low IDR (Figure 1: Patient A) to cases with a similar density in the epithelial and stromal aspects of the interface, represented by a high IDR (Figure 1: Patient B).

The results of the optimization experiments are presented in Table 1. The CD11c epithelial–stromal interface density ratio (IDR) achieved the highest concordance index (CI) on validation splits in the cross-validation. The CD8 IDR was the second-best feature; an IZ of 50 µm in thickness was found to be optimal for both cell subtypes. In general, the IDR performed better than cell densities in the entire interface zone (IZ) or in any other (epithelial or stromal) compartment of the tumor microenvironment.

Of note, for CD8, ICOS, and CD11c, the optimal IZ was narrower (50 µm, 40 µm, and 50 µm, respectively) than for CD20 and CD163 (150 µm and 140 µm, respectively). However, the latter two features showed a lower mean CI overall.

### 2.2. Univariate Cox Regression for Prediction of RFS

The results of the univariate Cox regression of features predictive of RFS with a *p*-value below 0.2, selected for the multiple Cox regression, are summarized in Table 2.

Patients with a history of positive re-TUR (repeated transurethral resection) had a significantly higher hazard ratio (HR) of 4.93 (*p*-value = 0.0001). Also, stage and grade emerged as potential prognostic factors. Patients with pT1 stage tumors (HR: 2.04, *p*-value = 0.0159) and high-grade tumors, according to the 1973 WHO classification (HR: 1.83, *p*-value = 0.0522), had a higher risk of recurrence, although the latter association was of borderline significance.

Several immune cell indicators revealed potential prognostic value. A low CD11c IDR (HR: 0.0012, *p*-value = 0.0002) and a low CD8 IDR (HR: 0.0379, *p*-value = 0.005) were associated with an increased risk of recurrence. Conversely, no significant association was observed for the density of these cells in neither epithelial nor stromal compartments. Stage and grade emerged as potential prognostic factors. Patients with pT1 stage tumors (HR: 2.04, *p*-value = 0.0159) and G3 grade tumors according to the 1973 WHO classification (HR: 1.83, *p*-value = 0.0522) had a higher risk of recurrence, although the latter association was of borderline significance.

We have identified TLSs in the TUR specimens of 100 patients (65.5%). The presence of TLSs did not show a statistically significant association with recurrence (HR: 1.69, *p*-value = 0.1033). Similarly, high-grade tumors, according to the 2004 WHO classification, and ICOS density in the epithelium, did not reach statistical significance (*p*-values > 0.1); nevertheless, we tested them in the multivariate analysis (see below).

### 2.3. Multiple Cox Regression

Multiple Cox regression analyses generated several models with moderate to good discriminative ability, with a mean CI ranging between 0.59 and 0.74. The best-performing model (see Table 3) consisted of two factors that were also the best-performing in the univariate Cox regression analysis, namely, a history of positive re-TUR and CD11c IDR, with a mean CI of 0.7427. Another strong model (CI > 0.7) included CD11c IDR and pT1 tumor stage. Interestingly, CD11c was included only in these two models, while re-TUR status was included only in the best-performing model.

The weaker models included all features with *p*-values < 0.05 from the univariate analysis, as well as the presence of TLSs, G3 tumor grade, CD163 IDR, and CD8 cell density in the epithelial compartment (the data are summarized in Table 4).

### 2.4. Kaplan–Meier RFS Analysis of the Selected Features

**Figure 2:**
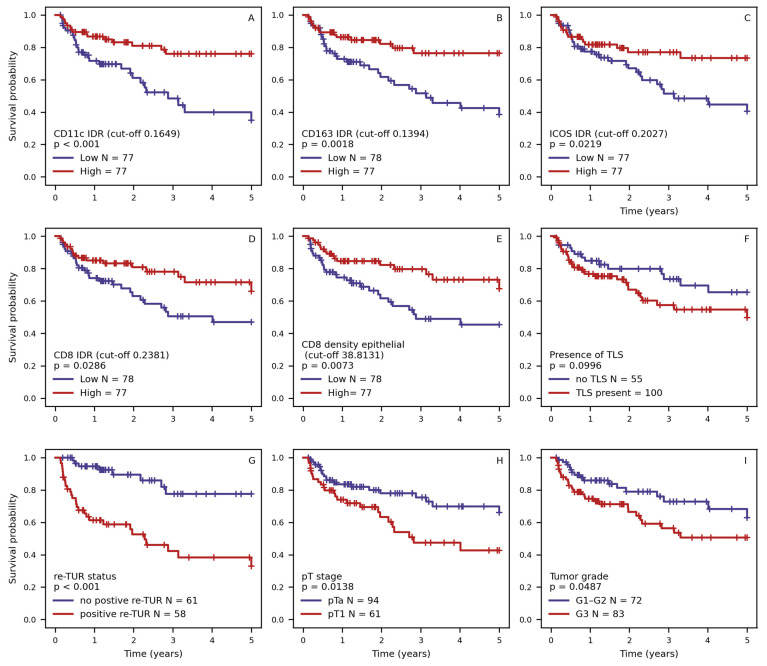
Kaplan–Maier RFS plots stratified on (**A**) CD11c interface density ratio (IDR) across epithelial–stromal interface, (**B**) CD163 IDR, (**C**) ICOS IDR, (**D**) CD8 IDR, (**E**) density of CD8 cells in epithelial compartment of interface zone, (**F**) presence of tertiary lymphoid structures (TLS), (**G**) presence of tumor in repeated TUR (re-TUR), (**H**) tumor stage, and (**I**) tumor grade. Continuous variables (**A**–**E**) are stratified according to median value of indicator.

The greatest difference between the risk groups of patients, similar to the results of the univariate Cox regression (Figure 2), was observed in cases with positive restaging transurethral resection (re-TUR) and CD11c IDR (*p* < 0.001). CD8 IDR, ICOS IDR, and tumor stage also showed statistically significant stratification of patient relapse risk (*p* < 0.05), which was also significantly associated with patients’ RFS in the univariate Cox regression. Additionally, G3 tumor grade, stratified CD163 IDR, and absolute CD8 cell density reached statistically significant (*p* < 0.05) stratification in the Kaplan–Meier analysis of RFS. This can be attributed to their non-linear effect on the risk of NMIPUC relapse. The presence of TLSs showed a tendency for prognostic stratification, but it did not reach statistical significance (*p* = 0.0996). On the other hand, other features such as WHO 2004 grade, ICOS density in the epithelial compartment, and CD8 density in the stromal compartment showed worse performance, paralleling the findings of the multivariate Cox regression analysis.

## 3. Discussion

In this study, we report computational models to predict NMIPUC relapse after BCG immunotherapy based on standard clinicopathologic factors and tumor-infiltrating immune cell densities and their density ratios across the epithelial–stromal interface. Remarkably, we found that in general, immune cell density ratio was more predictive of disease recurrence than absolute immune cell densities in any tumor tissue compartment or the entire interface zone. This confirmed our previously established prognostic role of the CD8 IDR [24]; nevertheless, the extended investigation has shown that the CD11c IDR has the best performance in the multivariate prognostic models, in combination with re-TUR and tumor stage. Meanwhile, CD20 and CD163 cell densities, or their ratio, did not reveal significant prognostic associations in our dataset.

The CD11c density ratio was the strongest prognostic feature from the immune tumor microenvironment features, followed by the CD8 IDR. CD11c is expressed in M1 polarized macrophages and was previously used in renal cell carcinoma [25], hepatocellular carcinoma [26], and carcinoma of the breast [30] to assess M1 macrophage infiltration. These studies revealed a higher CD11c-positive macrophage density associated with better patient outcomes. Dendritic cells also share the expression of CD11c, and multiplex imunotyping of CD11c-positive cell populations could improve the specificity of TAM assessment. However, according to the study by Ayari et al. [11], macrophages and dendritic cells have different distributions in NMIBC, and the narrow (50 µm) interface zone used in our study covers tissue area predominantly infiltrated by TAMs. Nevertheless, our findings might be impacted by minor components of CD11c-positive dendritic cells, since these cells play important roles in orchestrating antitumor immunity. Both CD11c-positive M1 macrophages and CD8-positive cytotoxic T lymphocytes are directly involved in antitumor immune response. Thus, their higher relative densities at the epithelial aspect of the interface can be interpreted as a representation of the higher intensity of immune response against the tumor. Conversely, other cells (M2 macrophages and B cells in our study) did not provide any prognostic value.

We found the density ratio of ICOS cells to be associated with RFS in the univariate analyses; ICOS expression can be present in both CD4+ helper T cells and CD8+ cytotoxic T cells. It is, therefore, difficult to interpret this finding; one could speculate that a higher ICOS IDR (a relatively higher intraepithelial ICOS-positive cell density) might reflect the increased proportion of ICOS+ cytotoxic T cells rather than helper T cells, taking into account the distribution of CD8 cells in our investigation. Studies subtyping ICOS cell populations preferably based on multiplex spatial immunoprofiling are needed to further investigate the role of ICOS expression. Nevertheless, the prognostic significance of the ICOS IDR in our study highlights the importance of these cells in the immune response in the context of BCG immunotherapy outcomes. Also, this may reveal the predictive potential of the ICOS IDR in the framework of upcoming ICOS/ICOSL immunotherapy options (REF).

Our study confirms the independent prognostic value of well-established clinicopathologic features—positive re-TUR, tumor stage, and tumor grade (according to the WHO 1978 classification)—to provide meaningful stratification of the recurrence risk [2].

The detection of TLSs was not significantly associated with RFS in our univariate analysis. Exploring the phenomenon of and variability in TLSs was not within the scope of the present study, where it was represented as a binary feature and therefore lacked prognostic value. The tissue sampling and intratumoral heterogeneity may also impact the detection and quantification of TLSs. Nevertheless, in our multivariate analysis, the TLS appeared in some weaker models, suggesting its relevance in the broader context of tumor microenvironment assessment. Studies investigating the composition of TLSs, similar to the study of Dijk et al. [29], might reveal more details on the importance of more specific subsets of TLSs.

Our study is based on a rather limited retrospective cohort of 155 patients from a single institution; thus, further prospective studies validating our findings in larger cohorts from multiple institutions are needed to support our findings. Additional studies, preferably based on multiplex immunofluorescence and spatial analytics, could enable more precise classification of tumor-infiltrating immune cell populations (T cell subclasses and dendritic cells), improving definitions of the prognostic features and adding new insights into the biological mechanisms behind them.

## 4. Materials and Methods

### 4.1. Patients

We collected clinical and pathological data of 230 BC patients who underwent adjuvant BCG immunotherapy at VUH SK from 2009 to 2020. For the study, we selected 165 BCG-treated (full 6-week induction course) NMIPUC patients, with available clinical, follow-up data and tumor TUR samples collected within one year prior to BCG induction. We limited the survival data to 5 years of follow-up to exclude cases that were more likely to be new primary tumors rather than “true recurrence”. We also collected data on repeated TUR performed in 121 patients. After preparing the IHC slides (see below), we excluded areas with co-agulation artifacts, and 10 cases were excluded from further analysis, resulting in the final set of 155 patients with sufficient tumor tissue quality. A summary of the patient clinical and pathologic data is provided in Table 5.

### 4.2. IHC Slide Preparation and Digitization

**Figure 3:**
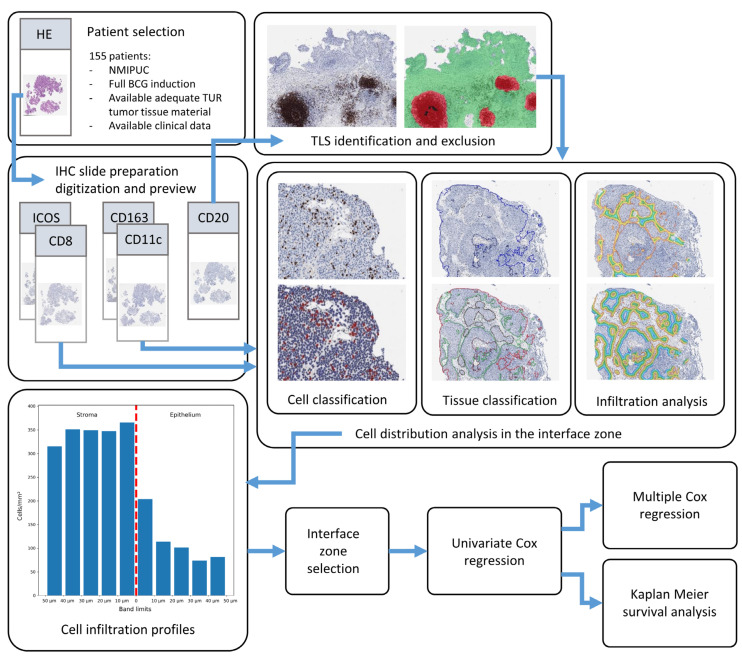
Study design chart. Patient selection and tissue selection were followed by IHC analysis and digitization. CD20 slides were then used for TLS identification, and images with excluded TLS areas, together with CD8, ICOS, CD11c, and CD163 images, underwent tissue and cell classification and infiltration analysis for assessment of cell infiltration profiles in epithelium–stroma interface. From resulting cell infiltration profiles, interface zone settings were optimized for all immune cell indicators, which were used for survival analysis to predict RFS.

A pathologist (JD) reviewed all of the archival material that was collected within a year prior to the initiation of BCG therapy. The pathologist then selected a single formalin-fixed paraffin-embedded tumor tissue block with the highest tumor grade and stage for each patient. Four-micrometer-thick tissue sections were used for IHC staining (Figure 3). We used a CD8 cytotoxic T cell marker (clone C8/144B, Dako, Glostrup, Denmark; dilution 1:100), B cell marker CD20 (clone L26, Dako, Glostrup, Denmark; dilution 1:500), M1 macrophage marker CD11c (clone 5D11, Leica biosystems, Deer Park, NY, USA; dilution 1:100), M2 macrophage marker CD163 (clone MRQ-26, Rocklin, CA, USA; dilution 1:50), and ICOS marker (clone D1K2T, Cell Signaling Technology, Danvers, MA, USA; dilution 1:500) for the identification of specific subpopulations of T cells. CD8, CD11c, and CD163 IHC were performed using Ventana Benchmark Ultra autostainer (Roche Diagnostics, Mannheim, Germany) and CD20 and ICOS using Dako Autostainer Link 48+ (Dako, Glostrup, Denmark). All slides were digitized at 20× magnification (0.5 µm per pixel) using an Aperio^®^ AT2 DX scanner (Leica Aperio Technologies, Vista, CA, USA).

### 4.3. Digital Image Analysis

We trained HALO^®^ AI (Indica Labs, Albuquerque, NM, USA) Densenet v2 classifiers for the identification of artifacts, mainly due to co-agulation resulting from the TUR procedure, and to segment epithelium and stroma compartments. In CD20 WSI, we also trained a classifier to segment TLSs and exclude them from further CD20 quantification in the remaining tissue. For the detection of CD8, CD20, and ICOS-positive lymphocytes, we used the HALO^®^ AI Multiplex IHC (Indica Labs, Albuquerque, NM, USA) module. Due to the irregularity of the cytoplasm in macrophages, the CD11c and CD163 macrophages were segmented by the HALO^®^ Nuclei seg (Indica Labs, Albuquerque, NM, USA) classifier followed by the HALO^®^ Nuclei Phenotyper (Indica Labs, Albuquerque, NM, USA) classifier.

### 4.4. Assessment of the Spatial Distribution of the Immune Cells

Spatial analysis of the immune cell infiltrates was performed within a 150 µm width epithelial–stromal interface zone extracted by the HALO^®^ AI Spatial Analysis module. Further, the width of the interface zone was decreased in 10 µm increments to search for the most informative width for the predictive modeling. We generated a set of indicators to characterize the cell infiltration (density in the entire interface zone and stromal and epithelial compartments) as well as cell distribution across the epithelial–stromal interface. The immune cell distributions were measured as the interface density ratio (IDR) across the epithelial–stromal interface, computed as the ratio of the cell density in the epithelial compartment and that in the stromal compartment of the interface zone. The presence of TLSs was expressed as a binary feature.

### 4.5. Statistical Analysis

To assess the most informative width of the interface zone for every indicator, we performed a univariate Cox regression with 50 iterations of 10-fold cross-validation to yield stable results, and used the mean concordance index (CI) across the validation splits as a performance metric.

The univariate Cox regression performed on the full dataset was used to select the most relevant features for the multiple Cox regression. Along with the clinical and pathology parameters, we tested tumor microenvironment aspects, including the presence of TLSs. We selected the features with a *p*-value of less than 0.2 and employed them in multiple Cox regression models. From all possible feature combinations, models with independent prognostic significance of features (*p* < 0.05 of all features in the model) were selected for further analysis. To assess the performance of these models, we used 10-fold cross-validation.

We performed a Kaplan–Meier survival analysis with the features included in selected multivariate models to assess their impact on patient risk stratification regarding NMIPUC recurrence. The statistical significance of the results was assessed using the log-rank test, with a *p*-value of 0.05 used as the threshold. For continuous variables, we used the median value as a cut-off to stratify the data.

## 5. Conclusions

Our investigation of the immune tumor microenvironment reveals the independent and superior prognostic value of pro-inflammatory CD11c macrophages with a relatively dense infiltration in the epithelial aspect of the tumor. We have shown the high predictive value of immune cell distribution in the tumor epithelium–stroma interface, assessed by an IDR indicator, outweighing the simple measurement of cell densities in the tumor. The strongest predictive value found in the cell populations directly involved in antitumor immunity (CD11c and CD8) highlights the IDR as a measure of immune system mobilization. These findings further support the value of spatial analytics in the tumor microenvironment while also providing useful means for NMIPUC patient risk stratification along with conventional clinicopathologic features.

## Figures and Tables

**Table 1:** The optimal interface zone width for the cell distribution features according to the mean concordance index (CI) in the cross-validation. Immune cell interface density ratio (IDR) across the epithelial–stromal interface.

IHC Marker	Feature	Optimal IZ Width (µm)	CI Mean (SD)
CD8	Total density	10	0.591 (0.157)
	Stromal density	10	0.574 (0.156)
	Epithelial density	130	0.62 (0.146)
	IDR	50	0.637 (0.146)
CD20	Total density	150	0.488 (0.162)
	Stromal density	150	0.503 (0.152)
	Epithelial density	140	0.51 (0.149)
	IDR	150	0.566 (0.151)
ICOS	Total density	10	0.503 (0.1417)
	Stromal density	10	0.504 (0.141)
	Epithelial density	30	0.513 (0.142)
	IDR	40	0.583 (0.162)
CD11c	Total density	10	0.518 (0.148)
	Stromal density	10	0.493 (0.147)
	Epithelial density	140	0.572 (0.157)
	IDR	50	0.64 (0.134)
CD163	Total density	40	0.51 (0.149)
	Stromal density	10	0.427 (0.132)
	Epithelial density	70	0.567 (0.142)
	IDR	140	0.603 (0.149)

**Table 2:** The results of the univariate Cox regression with a *p*-value lower than 0.2. IDR—immune cell interface density ratio across the epithelial–stromal interface. The stromal and epithelial density corresponds to the specific compartment of the interface zone or total area of the interface zone.

Feature	HR	*p*-Value
Positive re-TUR	4.9321	0.0001
CD11c IDR	0.0012	0.0002
CD8 IDR	0.0379	0.005
pT1	2.0445	0.0159
ICOS IDR	0.0768	0.0388
G3 grade (WHO 1973)	1.8254	0.0522
CD163 IDR	0.0712	0.0549
CD8 density total	0.9984	0.0648
CD8 density epithelial	0.996	0.0857
CD8 density stroma	0.9988	0.0988
Tertiary lymphoid structures	1.6915	0.1033
ICOS density epithelial	0.9963	0.1375
High grade (WHO 2004)	2.5873	0.1899

**Table 3:** Two Cox regression models with a concordance index > 0.7. IDR—interface density ratio across the epithelial–stromal interface.

Features	Hazard Ratio	95% CI	*p*-Value
Model: positive re-TUR + CD11c IDR			
Positive re-TUR	4.3411	1.9616–9.6072	<0.001
CD11c IDR	0.0282	0.00097–0.824	0.038
Model: pT1 stage + CD11c IDR			
pT1 stage	2.2524	1.2449–4.075	0.007
CD11c IDR	0.00067	0.000017–0.268	<0.001

**Table 4:** The results of the multivariate Cox regression with a *p*-value of individual features lower than 0.05. IDR—immune cell interface density ratio across the epithelial–stromal interface. TLS—the presence of tertiary lymphoid structures.

Model	AIC	Mean CI
CD11c IDR + positive re-TUR	257.8785	0.7427
CD11c IDR + pT1	338.225	0.703
CD8 IDR + TLS	352.2153	0.6449
ICOS IDR + G3	361.1213	0.6364
CD8 IDR + pT1	354.4931	0.6308
CD163 IDR + TLS	358.9342	0.6204
ICOS IDR + TLS + G3	356.6093	0.6143
CD163 IDR + G3	359.7073	0.6102
CD8 total density + TLS	360.0685	0.6084
ICOS IDR + pT1 + TLS	357.7706	0.6078
ICOS IDR + pT1	359.8559	0.6019
ICOS epithelial density + G3	360.1979	0.5974
CD163 IDR + pT1	358.9573	0.5972

**Table 5:** Summary of clinical and pathologic data.

Characteristic	Value (%)
Patients	155 (100%)
Age, years	
Median (range)	69.8 (33–89)
Gender	
Male	127 (81.9%)
Female	28 (18.1%)
RFS time, months	
Median (range)	16.3 (1.6–60)
Recurrences	46 (30%)
Tumor grade WHO 2004	
Low	12 (7.7%)
High	143 (92.2%)
Tumor grade WHO 1973	
G1	5 (3.2%)
G2	67 (43.2%)
G3	83 (53.6%)
pT stage	
Ta	94 (60.6%)
T1	61 (39.4%)
Carcinoma in situ association	8 (52%)
Positive re-TUR	55 (45.5%)
Recurrent tumor	45 (29%)
Multiple tumors	76 (49%)
Tumor size > 30 mm	43 (31.9%)
EORTC risk group	
Intermediate	71 (45.8%)
High	67 (43.2%)
Very high	5 (3.2%)

## Data Availability

The data presented in this study can be obtained from the authors upon request. These data are not available to the public due to permit restrictions.

## Supplementary Materials

The supporting information can be downloaded at: https://www.mdpi.com/article/10.3390/ijms25094776/s1.
