# Supplementary material for: Spatial Distribution of Macrophage and Lymphocyte Subtypes within Tumor Microenvironment to Predict Recurrence of Non-Muscle-Invasive Papillary Urothelial Carcinoma after BCG Immunotherapy
Source: Int J Mol Sci. 2024 Apr 27;25(9):4776. doi: 10.3390/ijms25094776 (PMC11084693; doi:10.3390/ijms25094776)
Supplement: Supplementary file 1 [file ijms-25-04776-s001.zip › ijms-2960023-supplementary.pdf]

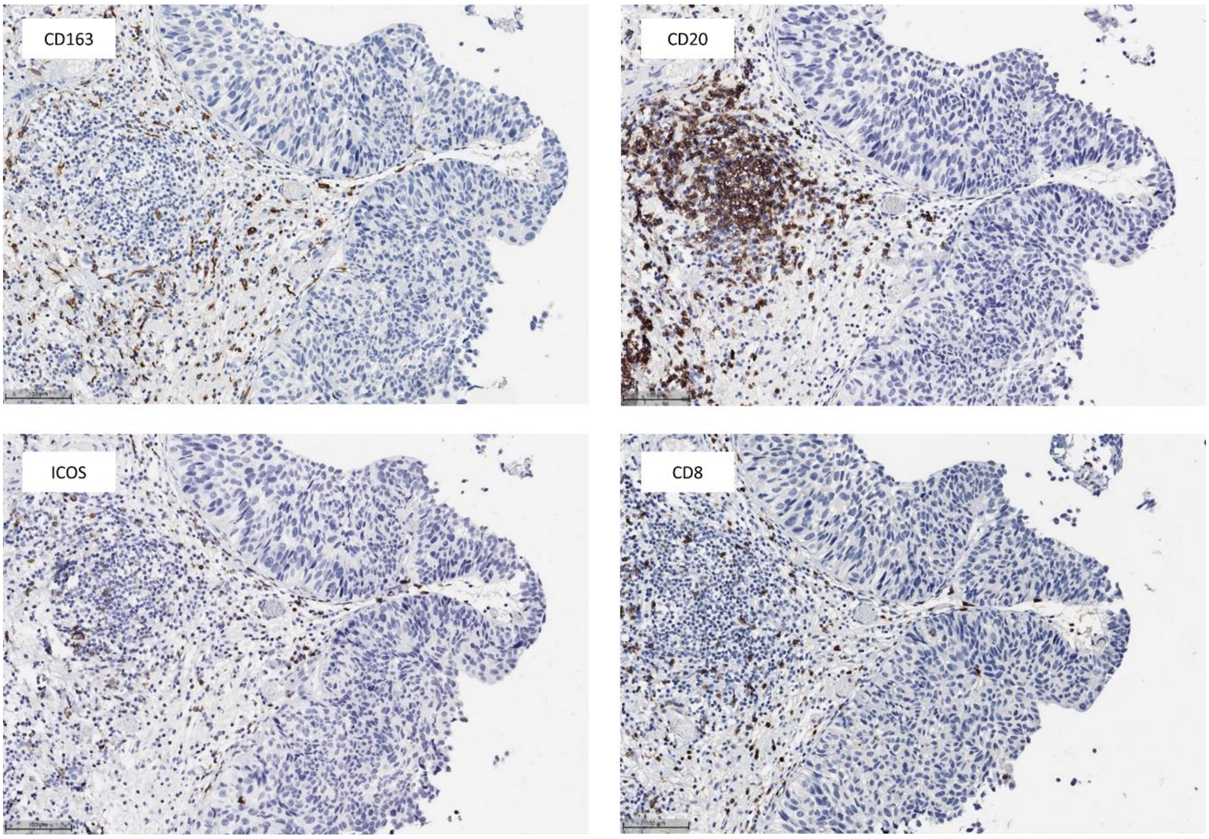

Supplementary Figure S1. Patient A: immunohistochemical reactions to CD163, CD20, ICOS, and CD8.

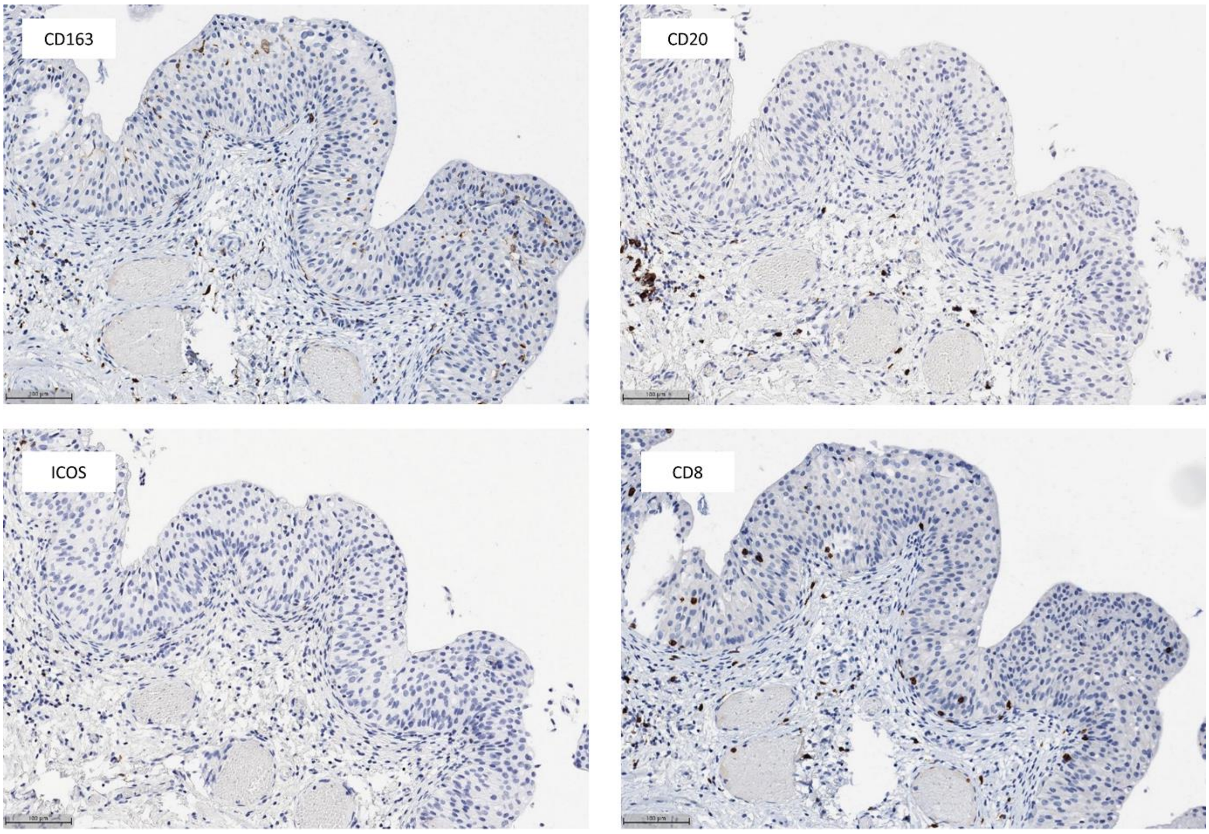

Supplementary Figure S2. Patient B: immunohistochemical reactions to CD163, CD20, ICOS, and CD8.
